# Relationship between intramuscular fat content in *longissimus thoracis* and hair fatty acids in finishing crossbred bulls

**DOI:** 10.1038/s41598-025-29857-8

**Published:** 2025-11-27

**Authors:** Ramona Wulf, Danny Arends, Ariane Boldt, Sabine Schmidt, Steffen Maak, Dirk Dannenberger, Gudrun A. Brockmann

**Affiliations:** 1 Hochschule Neubrandenburg, University of Applied Science, Brodaer Str. 2, 17033 Neubrandenburg, Germany; 2 Albrecht Daniel Thaer-Institute, Humboldt-Universität zu Berlin, Unter den Linden 6, 10099 Berlin, Germany; 3 Department of Applied Sciences, Northumbria University, Newcastle upon Tyne, UK; 4 Institute of Livestock Farming, Mecklenburg-Vorpommern Research Centre for Agriculture and Fisheries, 18196 Dummerstorf, Germany; 5 RinderAllianz, Am Bullenberg 1, 17348 Woldegk, Germany; 6 Research Institute for Farm Animal Biology (FBN), Wilhelm-Stahl-Allee 2, 18196 Dummerstorf, Germany

**Keywords:** Meat quality, Estimation, Non-invasive biomarker, Essential fatty acids, Cattle, Fat metabolism, Predictive markers

## Abstract

Intramuscular fat (IMF) content below 6% in live cattle is difficult to estimate accurately. Therefore, this study tested whether fatty acids in the IMF of LT and hair correlate to each other, and whether fatty acids can be used to predict IMF. Forty-four finishing crossbred bulls from two farms were examined. Each bull had a hair sample collected before slaughter, and two days post-slaughter, LT muscle samples were collected to assess IMF (range: 2.3–6.6%) and fatty acids. Predictions of IMF from hair fatty acids were developed using linear, non-linear and non-parametric approaches. Nineteen congruent fatty acids could be detected in hair and LT. Although proportions varied, lauric and oleic acids showed positive correlations between LT and hair (*r* = 0.48–0.49; *P* ≤ 0.001). Lauric acid in hair showed also a consistently prediction of IMF and moderate rank-based association between predicted and observed IMF across all tested prediction models. For the first time, the study presents evidence that hair fatty acids taken shortly before slaughter provide significant information about the IMF in bulls. To use this information for breeding or feeding strategies, it would be necessary to extend hair sampling by testing earlier stages in life.

**Supplementary Information:**

The online version contains supplementary material available at 10.1038/s41598-025-29857-8.

## Introduction

The intramuscular fat (IMF) content of cattle is a key determinant of meat quality. Higher IMF content positively influences flavor, juiciness and tenderness of meat. The average IMF content of European breeds ranges from 0.6 to 4.8%^1–4^. Depending on the breed, the IMF content can vary widely, for example from 1% in White-Blue Belgian to 37% in Wagyu. In addition to breed, the diet is the key factor determining the IMF besides age at weaning, sex, castration, age at slaughter and weight at slaughter^5,6^. The optimal IMF content for consumers is recommended between 3% to reach an acceptable palatability and 7.3% to ensure nutritional merits^7^.

The exact IMF content of a muscle is determined by chemical analysis after slaughter. To improve the production of high-quality beef with high IMF content, it would be desirable to know the individual IMF content during the finishing period prior to slaughter. This would allow producers to make decisions about increasing the intensity of fattening or extending the finishing period.

The ultrasound method is being tested to determine the IMF content of live animals. This method is non-invasive and fast, but the method is not very reliable in breeds with low IMF content. The reliability and accuracy of IMF measurement depends mainly on the type of ultrasound equipment and transducer used, the movements of the animal and the experience of the person performing the measurement^8^.

We propose the analysis of fatty acids in the hair of finishing bulls as another strategy to access the IMF content of live animals. Hair samples have two major advantages. Firstly, hair samples can be collected non-invasively and secondly, hair lipids contain fatty acids that are also present in the IMF. Fatty acids found in the IMF and hair include palmitic, stearic and oleic acid, which are the major fatty acids of the IMF, the essential fatty acids linoleic and *alpha*-linolenic acid and the *de novo* biosynthesized fatty acids lauric and myristic acid. In the *longissimus thoracis* (LT) moderately high correlations were shown between total IMF content and both oleic (*r* = 0.5) and linoleic acid (*r* = -0.6) percentages^9^. Furthermore, positive correlations were found between hair fatty acids and the energy metabolism during early lactation in cows^10–12^.

Recently, the total IMF content of lambs has been correlated with fatty acids in wool. In particular capric, lauric and myristic acid were positive correlated and oleic acid was negatively correlated to IMF content^13^. However, it is not known whether hair fatty acids of bulls are correlated to IMF content. Therefore, this study aimed at finishing bulls to test (1) if fatty acids in the IMF of LT and hair correlate to each other, and (2) if individual fatty acids can use to predict IMF content. The study was carried out on LT as a valuable muscle and on the hair of the skin above the muscle. Fatty acids were expressed as concentrations as well as percentages. We expected that individual fatty acids in hair are related to their concentration and percentage as well as to IMF in LT and are therefore useful for predicting IMF in crossbred bulls.

## Results

### Comparison of fat deposition between farms (UCKxBEEF, farms 1 and 2) and crossbreeds (UCKxBEEF vs. WBBxSBT, farm 2)

The average age of UCKxBEEF bulls at the time of slaughter was 34 days lower in farm 1 than in farm 2. The age range at which the UCKxBEEF bulls were slaughtered varied widely on farm 1, ranging from 525 to 626 days. Of the UCKxBEEF bulls in farm 2, 11 were slaughtered at a similar age of 619 ± 14 days, two were younger (515 and 517) and two were older (664 and 691). At farm 2, UCKxBEEF bulls were slaughtered 26 days earlier than WBBxSBT bulls. Despite the cross and farm differences in age, carcass weights were similar on both farms, ranging from 357 kg to 521 kg on farm 1 and from 387 kg to 492 kg on farm 2. Weights were also similar between crossbreeds on farm 2 (Table 1).

**Table 1:** Animal traits, ultrasound measurement and meat intramuscular fat content of bulls from farm 1 and farm 2 (mean ± SE).

	Farm 1	Farm 2		*P*-values^1^	
	UCKxBEEF (*n* = 21)	UCKxBEEF (*n* = 15)	WBBxSBT (*n* = 8)	Farm 1 UCKxBEEF vs. Farm 2 UCKxBEEF	Farm 2 UCKxBEEF vs. WBBxSBT
Animal traits					
Age at slaughter (days)	574 ± 6.5	607 ± 11.4	633 ± 5.7	0.003	0.042
Carcass weight (kg)	432 ± 9.4	438 ± 5.9	431 ± 13.1	0.526	0.875
Ultrasound measurements (12./13. rib, 2 days a.m.)					
Muscle area (cm²)	99.1 ± 2.4	107.6 ± 2.6	108.3 ± 2.6	0.034	0.627
Fat layer (cm)	0.8 ± 0.04	0.7 ± 0.03	0.5 ± 0.04	0.318	0.089
Measurements of longissimus thoracis (5./6. rib, 2 days p.m.)					
NIRS^2^-IMF (%)	4.0 ± 0.3	5.3 ± 0.3	3.8 ± 0.4	0.002	0.007

^1^*P*-values from Wilcoxon-test; ^2^ NIRS - Near-infrared spectroscopy.

**Fig. 1:**
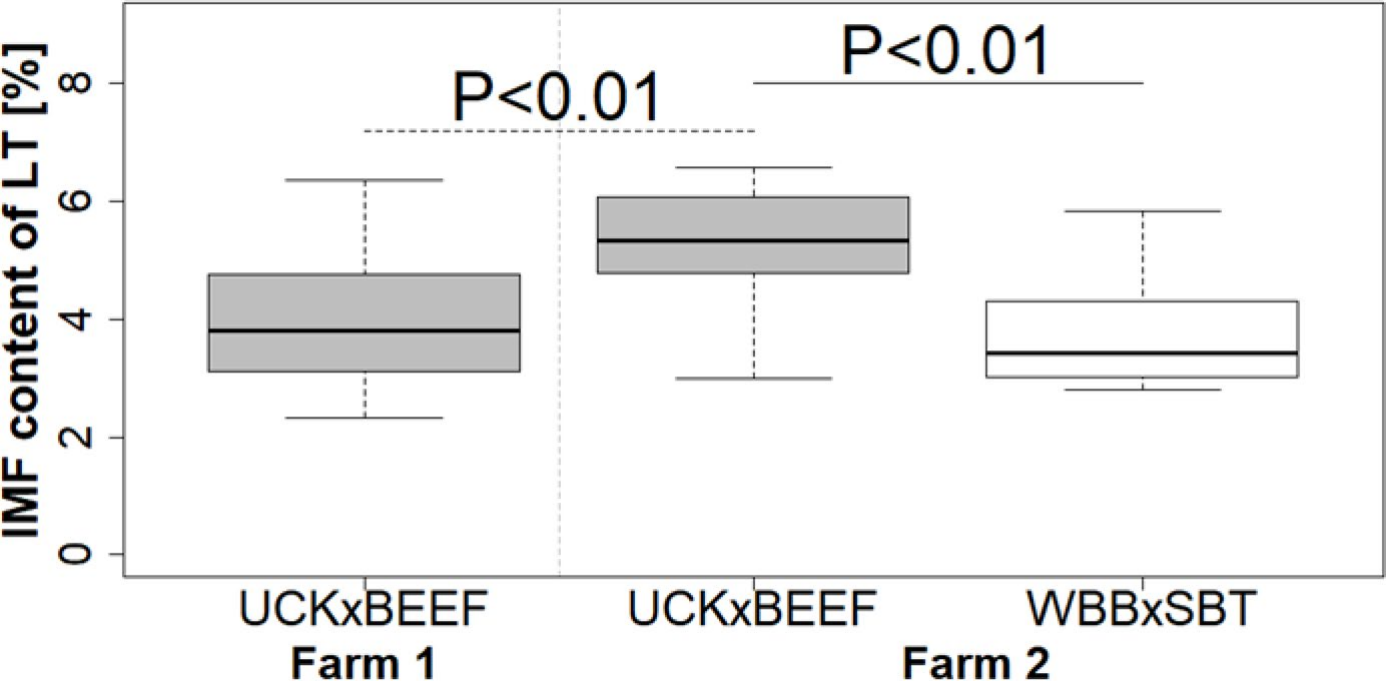
Intramuscular fat (IMF) content of *longissimus thoracis* (LT) based on farm and crossbreed; farm 1 (gray filled square—UCKxBEEF bulls, *n* = 21) and farm 2 (gray filled square——UCKxBEEF bulls, *n* = 15; open square—WBBxSBT bulls, *n* = 8); black dotted line—significant difference between UCKxBEEF bulls from farm 1 and 2, black line—significant difference between UCKxBEEF and WBBxSBT bulls from farm 2.

The LT had an IMF content of 2.3% to 6.6% in the different crossbreed x farm relations. Bulls from UCKxBEEF in farm 2 showed an improved meat quality of LT, characterized by a higher IMF content compared to the meat produced by UCKxBEEF bulls from farm 1 (mean farm 1: 4.0%; farm 2: 5.3%; min - max farm 1: 2.3–6.4%; farm 2: 3.0–6.6%). On farm 2, UCKxBEEF bulls had 1.4 times higher IMF compared to WBBxSBT bulls (*P* < 0.03, Fig. 1; Table 1).

In LT, the percentage of oleic acid was 0.9-times lower in the UCKxBEEF bulls of farm 1 compared to farm 2 (*P* < 0.01, Supplemental material Tables 1 and 2). Furthermore, the percentages of capric, lauric and myristic acid as well as odd and branched chain fatty acids (tridecanoic, pentadecanoic, *iso*-myristic and *anteiso*-pentadecanoic acid) were higher in UCKxBEEF bulls in farm 1 than in farm 2, while the percentages of palmitic, stearic and linoleic acid in LT did not differ (*P* > 0.05). Among farm 2 bulls, those from the UCKxBEEF cross had higher oleic acid and lower capric, lauric, linoleic, tridecanoic, *iso*-myristic and *anteiso*-pentadecanoic acid in LT than those from the WBBxSBT cross (*P* < 0.05).

**Table 2:** Comparison of fatty acid pattern in LT and hair (% of total fatty acids) within farm.

	Farm 1				Farm 2							
	UCKxBEEF (*n* = 21)				UCKxBEEF (*n* = 15)				WBBxSBT (*n* = 8)			
	LT	Hair	SEM	*P-Value*	LT	Hair	SEM	*P-Value*	LT	Hair	SEM	*P-Value*
	EMM	EMM			EMM	EMM			EMM	EMM		
SFA	49.30	86.05	0.83	< 0.001	47.59	77.74	1.40	< 0.001	47.49	88.73	0.88	< 0.001
C10:0 (capric)	0.10	6.58	0.21	< 0.001	0.08	5.31	0.28	< 0.001	0.09	7.20	0.17	< 0.001
C12:0 (lauric)	0.11	4.03	0.11	< 0.001	0.08	3.47	0.16	< 0.001	0.10	4.67	0.07	< 0.001
C14:0 (myristic)	4.16	40.22	1.10	< 0.001	3.43	34.44	1.49	< 0.001	3.36	45.63	1.13	< 0.001
C16:0 (palmitic)	27.10	16.66	0.57	< 0.001	26.42	17.58	0.89	< 0.001	26.11	14.32	0.79	< 0.001
C18:0 (stearic)	17.55	10.31	0.49	< 0.001	17.40	10.32	0.56	< 0.001	17.58	8.69	0.58	< 0.001
OBCFA	2.29	2.06	0.08	0.049	1.81	4.13	0.14	< 0.001	1.94	3.81	0.14	< 0.001
C15:0 (pentadecanoic)	0.40	0.54	0.02	< 0.001	0.28	1.81	0.08	< 0.001	0.30	1.03	0.03	< 0.001
C17:0 (heptadecanoic)	0.90	0.33	0.02	< 0.001	0.85	0.41	0.03	< 0.001	0.87	0.39	0.03	< 0.001
C14:0*iso* (*iso*-myristic)	0.13	0.66	0.04	< 0.001	0.08	1.15	0.06	< 0.001	0.09	1.62	0.11	< 0.001
C15:0a.i. (*anteiso-*pentadecanoic)	0.22	0.26	0.01	0.089	0.16	0.20	0.01	0.001	0.18	0.27	0.01	< 0.001
MUFA	42.02	8.68	0.79	< 0.001	44.88	13.67	1.17	< 0.001	41.87	5.57	0.59	< 0.001
*c*9-C18:1 (oleic)	36.83	5.41	0.64	< 0.001	40.22	9.18	1.02	< 0.001	36.57	3.71	0.43	< 0.001
*c11*-C18:1	1.33	2.47	0.17	< 0.001	1.69	3.42	0.25	< 0.001	1.80	1.35	0.15	0.067
PUFA	6.40	3.21	0.37	< 0.001	5.72	4.46	0.41	0.048	8.70	1.89	0.50	< 0.001
C18:2*n*-6 (linoleic)	3.92	3.08	0.27	0.037	3.66	4.15	0.31	0.285	5.63	1.77	0.34	< 0.001
C18:3*n*-3 (ALA)	0.30	0.17	0.02	< 0.001	0.35	0.31	0.05	0.597	0.37	0.11	0.02	< 0.001

EMM - estimated marginal means; SEM -standard error of the mean.

SFA = sum of saturated fatty acids (C10:0, C12:0, C14:0, C16:0, C18:0, C20:0, C22:0, C24:0, C26:0), in LT C24:0 and C26:0 not detected; OBCFA = sum of odd and branched chain fatty acids (C11:0, C13:0, C15:0, C17:0, C21:0, C23:0, C13:0a.i., C14:0*iso*, C15:0*iso*, C15:0a.i., C16:0*iso)*, in hair C11:0, C23:0, C13:0a.i., C15:0*iso* and C16:0*iso* not detected; MUFA = sum of monounsaturated fatty acids (C14:1, C16:1, C17:1, *t*9-C18:1, *t11*-C18:1, *c*9-C18:1, *c*11-C18:1, C20:1, C22:1, C24:1), in LT C24:1 not detected; in hair C14:1, C17:1, *t*9-C18:1 and *t11*-C18:1 not detected; PUFA = sum of polyunsaturated fatty acids (*t-*C18:2, C18:2*n*-6, C18:3*n*-6, C18:3*n*-3, *c9*,*t11*-CLA, C20:2*n*-6, C20:3*n*-9, C20:3*n*-6, C20:4*n*-6, C20:5*n*-3, C22:4*n*-6, C22:2*n*-6, C22:6*n*-3), in hair *t-*C18:2, C18:3*n*-6, *c9*,*t11*-CLA, C20:2*n*-6, C20:3*n*-9, C20:3*n*-6, C20:4*n*-6, C20:5*n*-3, C22:4*n*-6, C22:2*n*-6, C22:6*n*-3 not detected.

**Fig. 2:**
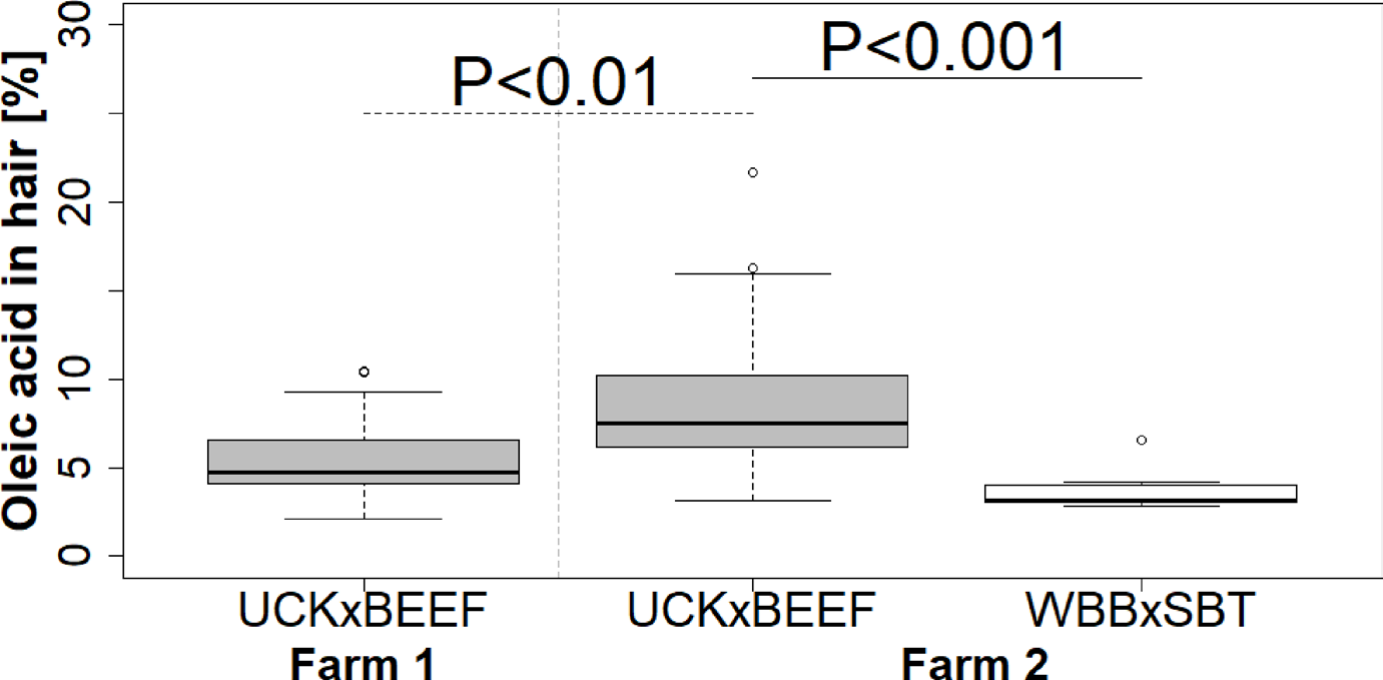
Oleic acid contents [%] in hair based on farm and crossbreed; farm 1 (gray filled square—UCKxBEEF bulls, *n* = 21) and farm 2 (gray filled square—UCKxBEEF bulls, *n* = 15; open square—WBBxSBT bulls, *n* = 8); black dotted line—significant difference between UCKxBEEF bulls from farm 1 and 2, black line—significant difference between UCKxBEEF and WBBxSBT bulls from farm 2.

Similar to the muscle, the percentage of oleic acid in the hair was significantly lower in UCKxBEEF bulls from farm 1 than in UCKxBEEF bulls from farm 2, even if the difference in the hair was more pronounced (*P* = 0.004; Fig. 2; Supplemental material Tables 1 and 2). Additionally, percentages of capric, lauric and myristic acid in hair were higher in UCKxBEEF bulls in farm 1, while percentages of palmitic and stearic acid in hair were unchanged between UCKxBEEF bulls from both farms. In contrast to the fatty acid percentage in muscle, the tridecanoic, pentadecanoic, *iso*-myristic and linoleic acid percentages in hair were significantly lower in UCKxBEEF bulls from farm 1 compared to farm 2 (*P* < 0.05).

In farm 2, the UCKxBEEF bulls had higher percentages of oleic acid and lower percentages of capric, lauric, myristic and *iso*-myristic acid and *anteiso*-pentadecanoic acid in the IMF of the muscle and in the hair compared to WBBxSBT bulls (*P* < 0.05). The linoleic acid percentage in the IMF of the muscle was lower in UCKxBEEF bulls than in WBBxSBT bulls, but in hair it was opposite, with higher percentages in UCKxBEEF compared to WBBxSBT.

### Comparison of the patterns of fatty acids between hair and longissimus thoracis (LT) in UCKxBEEF and WBBxSBT bulls

The composition of the fatty acids that were detectable in LT and hair differed significantly. Nineteen fatty acids were detected in both LT and hair. Additional fatty acids that have only detected in the muscle are the longer chain PUFAs (> 20 carbons), such as arachidonic acid and eicosapentaenoic acid. The long chain SFAs lignoceric acid and cerotic acid as well as the MUFA erucic acid were only found in the hair. In the LT of the different crossbreeds, the sum of SFAs (47–49%) and the sum of MUFAs (42–45%) was the same, in the hair most fatty acids were SFAs (78–89%, *P* < 0.05, Tables 2 and 3).

**Table 3:** Comparison of fatty acid pattern in LT and hair (mg/ 100 g tissue) within farm.

	Farm 1				Farm 2							
	UCKxBEEF (*n* = 21)				UCKxBEEF (*n* = 15)				WBBxSBT (*n* = 8)			
	LT	Hair	SE	*P-Value*	LT	Hair	SE	*P-Value*	LT	Hair	SE	*P-Value*
	EMM	EMM			EMM	EMM			EMM	EMM		
Total fatty acids	2925.66	419.92	145.33	< 0.001	4105.99	482.31	217.28	< 0.001	2351.22	444.41	179.31	< 0.001
SFA	1443.00	362.98	74.78	< 0.001	1979.40	367.45	121.33	< 0.001	1130.33	394.62	103.10	0.001
C10:0 (capric)	2.93	28.34	1.84	< 0.001	3.36	26.73	3.25	< 0.001	2.10	32.20	1.73	< 0.001
C12:0 (lauric)	3.07	17.35	1.11	< 0.001	3.12	17.36	2.11	< 0.001	2.32	20.84	0.94	< 0.001
C14:0 (myristic)	124.26	172.76	14.15	0.024	144.77	172.70	23.12	0.366	83.33	204.19	15.84	0.001
C16:0 (palmitic)	793.66	68.25	41.85	< 0.001	1100.06	75.15	66.04	< 0.001	625.29	63.04	61.30	< 0.001
C18:0 (stearic)	511.50	41.62	25.09	< 0.001	720.32	43.98	42.01	< 0.001	411.86	37.94	30.53	< 0.001
OBCFA	65.80	8.61	2.85	< 0.001	74.85	20.77	5.10	< 0.001	45.29	17.11	3.40	0.001
C15:0 (pentadecanoic)	11.50	2.27	0.54	< 0.001	11.82	9.40	1.56	0.217	7.15	4.59	0.67	0.030
C17:0 (heptadecanoic)	26.51	1.35	1.39	< 0.001	35.29	1.71	2.20	< 0.001	20.25	1.72	1.45	< 0.001
C14:0*iso*	3.63	2.81	0.27	0.046	3.05	5.83	0.85	0.029	2.07	7.36	0.73	0.001
C15:0a.i.	6.27	1.07	0.27	< 0.001	6.64	0.95	0.44	< 0.001	4.31	1.21	0.32	< 0.001
MUFA	1245.72	34.74	70.99	< 0.001	1830.86	69.06	89.05	< 0.001	982.10	24.26	71.53	< 0.001
*c*9-C18:1 (oleic)	1092.68	21.81	62.92	< 0.001	1644.55	50.30	81.83	< 0.001	860.06	16.23	65.71	< 0.001
*c11*-C18:1	38.51	9.67	2.11	< 0.001	67.79	14.15	3.07	< 0.001	41.86	5.79	2.80	< 0.001
PUFA	171.14	13.59	3.45	< 0.001	220.87	25.02	7.82	< 0.001	193.49	8.42	4.71	< 0.001
C18:2*n*-6 (linoleic)	105.02	13.02	2.92	< 0.001	141.85	22.86	6.53	< 0.001	125.59	7.91	4.02	< 0.001
C18:3*n*-3 (ALA)	8.15	0.74	0.34	< 0.001	13.80	2.16	1.00	< 0.001	8.23	0.51	0.30	< 0.001

See legend in Table 2.

Among the SFAs, especially the medium chain fatty acids up to 14 carbons, such as capric, lauric and myristic acid, had 1.2- to 15.3-times higher concentrations in hair compared to LT (mg/100 g tissue). These three fatty acids accounted for only 4% of total fatty acids in LT, but for about the half of total fatty acids in the hair. The main saturated fatty acids in the muscle are palmitic and stearic acid. The percentages of these two fatty acids in the muscle was high with 44%, while the percentages in the hair was 23–27%.

The most abundant MUFA in the muscle is oleic acid. Its percentages in LT were 4.4- to 9.9-times higher than in hair (%; *P* < 0.001). With respect to the essential fatty acids linoleic and *alpha*-linolenic acid, the concentrations (mg/100 g tissue) were 6.2- to 16.1-times higher in LT compared to hair.

### Correlation between intramuscular fat content and fatty acids in longissimus thoracis and hair

Correlation analyses were performed to determine the relationships (1) between fatty acids of LT and fatty acids of hair and (2) between IMF content of LT and fatty acids of the hair. For this analysis, correlation coefficients were calculated for the total dataset across all bulls and for the farm- and crossbreed-specific data. In the total dataset, higher percentages or concentrations of MUFA in LT were correlated to higher MUFA in hair (%, mg/ 100 g; *r* = 0.54; *P* < 0.001, Table 4). Within farms and within crossbreeds the correlations showed in the same direction, but they were not significant (Supplemental material Tables 3 and 4). When the percentages and concentration of oleic acid in LT (%, mg/ 100 g) were high, the values for oleic acid in the hair were also higher.

**Table 4:** Spearman’s correlation coefficient between fatty acids in LT and hair in the total dataset (*n* = 44).

	Fatty acids in LT and hair	
	*r*	*P*-Value
Fatty acid percentages (% of total fatty acids)		
SFA		
C12:0 (lauric)	0.48	0.001
OBCFA	-0.59	< 0.001
C15:0 (pentadecanoic)	-0.65	< 0.001
C14:0*iso* (*iso*-myristic)	-0.52	< 0.001
MUFA	0.54	< 0.001
*c*9-C18:1 (oleic)	0.49	0.001
PUFA	-0.52	< 0.001
C18:2*n*-6 (linoleic)	-0.51	< 0.001
Fatty acid concentration (mg/ 100 g tissue)		
OBCFA		
C14:0*iso*	-0.33	0.027
MUFA	0.54	< 0.001
*c*9-C18:1 (oleic)	0.52	< 0.001
*c11*-C18:1	0.49	0.001

See legend in Table 2.

Highly significant positive correlations were found between the percentages of lauric acid in LT and hair in the analysis of the total dataset (*r* = 0.48; *P* = 0.001) and when farm 1 was analyzed separately (UCKxBEEF, *r* = 0.49; *P* < 0.05). In the total dataset, we also found that higher percentages of linoleic and pentadecanoic acid in the hair were negatively correlated to their percentages in LT (-0.65 ≤ *r* ≤ -0.51; *P* < 0.001).

In the total dataset, with increasing IMF, the sum of MUFAs (%, mg/ 100 g) increased in LT (0.35 ≤ *r* ≤ 0.94; *P* ≤ 0.001) and in hair (0.45 ≤ *r* ≤ 0.47; *P* < 0.01; Supplemental material Tables 5 and 6). Especially, oleic acid in LT and in hair (%, mg/ 100 g) were higher when IMF content was higher.

**Table 5:** Performance of hair fatty acids to predict IMF (%) of *longissimus thoracis* using linear regression (LR), quantile regression (QR), generalized additive model (GAM) and random forest (RF).

	Prediction models							
	LR		QR		GAM		RF	
	MSE	r_S_	MSE	r_S_	MSE	r_S_	MSE	r_S_
Fatty acid percentages (% of total fatty acids)								
C10:0 (capric)	1.32	0.23	1.26	0.27	1.28	0.37	1.58	0.15
C12:0 (lauric)	1.30	0.25	1.26	0.24	1.13	0.40	1.44	0.26
C14:0 (myristic)	1.33	0.12	1.26	0.25	1.36	0.26	1.65	0.09
C15:0 (pentadecanoic)	1.34	0.13	1.41	0.27	1.47	0.21	1.49	0.15
C15:0a.i.	1.30	0.11	1.34	0.32	1.29	0.28	1.75	-0.02
*c*9-C18:1 (oleic)	1.26	0.33	1.27	0.24	1.52	0.15	1.52	0.28
*c11*-C18:1	1.34	0.09	1.30	0.26	1.45	0.10	1.71	0.16
C18:2*n*-6 (linoleic)	1.22	0.38	1.22	0.30	2.25	0.22	1.59	0.23
C18:3*n*-3 (ALA)	1.20	0.37	2.86	0.24	5.45	0.32	1.28	0.28
Sum of fatty acids								
SFA	1.28	0.31	1.26	0.25	1.59	0.12	1.40	0.21
OBCFA	1.32	0.11	1.26	0.24	1.51	0.08	1.74	-0.03
MUFA	1.31	0.29	1.26	0.26	1.37	0.24	1.53	0.17
PUFA	1.21	0.39	1.26	0.29	2.41	0.22	1.60	0.22
Fatty acid concentration (mg/ 100 g tissue)								
C15:0 (pentadecanoic)	1.25	0.19	1.26	0.27	1.71	0.02	1.65	0.03
*c*9-C18:1 (oleic)	2.26	0.24	1.26	0.25	3.11	0.25	1.58	0.14
*c11*-C18:1	1.24	0.32	1.26	0.24	1.36	0.15	1.68	0.11
C18:2*n*-6 (linoleic)	1.41	0.27	1.27	0.24	7.92	0.29	1.67	0.03
C18:3*n*-3 (ALA)	2.55	0.27	6.03	0.29	18.11	0.23	1.58	0.02
Sum of fatty acids								
MUFA	1.65	0.25	1.25	0.25	6.79	0.21	1.53	0.26
PUFA	1.49	0.26	1.26	0.25	8.15	0.30	1.67	0.10

LR - linear regression; QR - quantile regression; GAM - generalized additive model; RF - randomForest; MSE - Mean Squared Error; r_S_ - Spearmann correlation coefficient; for sum of fatty acids; for sum of fatty acids see legend in Table 2.

The IMF content is positively correlated with the sum of PUFAs in the hair (mg/ 100 g; *r* = 0.31; *P* < 0.05, Supplemental material Table 6). Particularly, linoleic acid and *alpha*-linolenic acid are higher in the hair with increasing IMF (mg/ 100 g; 0.31 ≤ *r* ≤ 0.46; *P* < 0.001). Furthermore, the IMF content is negatively correlated with the sum of SFAs in the hair. This is particularly observed for the individual SFAs capric, lauric and myristic acid (%; -0.47 ≤ *r* ≤ -0.30; *P* ≤ 0.05). However, the correlation coefficients between IMF content and concentrations of capric, lauric and myristic acid in hair were not significant.

## Prediction of intramuscular fat content based on fatty acids in hair

To predict the IMF content in LT, we tested whether individual fatty acids are suitable for predicting IMF by testing linear, nonlinear, and nonparametric approaches. The prediction modeling revealed that prediction of IMF in LT could be performed from individual hair fatty acids (Table 5). Predictive performance revealed that models including lauric acid (%) consistently produced IMF (%) prediction with good accuracy and moderate precisions. Using lauric acid (%) as predictor, the agreement between the observed and predicted IMF was lowest in the quantile regression (r_S_=0.24) and highest in the generalized additive model (r_S_=0.40). Using the generalized additive model, the lowest MSE across all tested models was also achieved (MSE < 1.13%). Using oleic acid (%) as predictor, best predictive performance was calculated using the linear regression, quantile regression and randomForest (1.26 ≤ MSE ≤ 1.52%; 0.24 ≤ r_S_ ≤ 0.33). In an interesting range of the IMF (defined as IMF between 3.5 and 4.5%; *n* = 11), the mean of the observed IMF was 4.01 ± 0.33% (mean ± standard deviation (SD)). The mean of the predicted IMF was 4.04 ± 0.36% (mean ± SD) using the generalized additive model with lauric acid as predictor. As such that 95% of the predictions were between 3.3 and 4.7% IMF.

## Discussion

To market high-quality beef, it is crucial to slaughter bulls at the best time point. Since the IMF content is a key parameter of high meat quality, the knowledge of IMF content in live animals would allow to influence the muscle composition and finally to ensure consistent beef quality for the consumers. To date, the ultrasound measurement is the only method for non-invasive determination of the IMF content in live animals. A drawback of the ultrasound is that fat content estimates are reliable only if the total fat content is higher than 6%^14,15^. Since the IMF content in beef from bulls lies on average between 0.6 and 4.8%, an alternative strategy is needed to assess the IMF content in live animals during the fattening period before slaughtering. Therefore, we examined in the current study if fatty acids in the hair of finishing bulls taken shortly before slaughter correlate with the IMF content of LT as a particularly valuable muscle and if individual fatty acids are potential indicators to predict IMF content in live bulls.

The use of fatty acids in hair can provide predictions for IMF content in live bulls which varied in IMF between 2.3 and 6.6%. In this range of IMF content, the ultrasound measurement is not reliable. Since IMF is a key determinant of meat quality, the development of predictive equations for estimating the meat quality of live animals is of great importance for optimizing meat production and marketing. For example, the prediction of IMF could be used to phenotype offsprings of sires at several points during in the fattening period. Simple and non-invasive indicators that are reliable even at moderate IMF contents and can be determined several times on live animals, such as hair fatty acids, are therefore necessary.

To our knowledge, this is the first study in which predictions based on fatty acids from bulls’ hair were tested using different modeling approaches, such as linear, non-parametric and non-linear regression. Especially percentages of lauric and oleic acid in hair can predict IMF with good accuracy and moderate rank-based association between predicted and observed IMF consistently across linear, non-parametric and non-linear approaches. In this study, the lowest MSE of 1.13% is quite high. However, it is due to the low sample size. Between 3.5 and 4.5% IMF, predictions using the generalized additive model and lauric acid as predictor are better. The mean of the predicted IMF was close to the observed IMF. The standard deviation of the prediction was the same, which means that the prediction did not reduce the biological variance within this IMF range. Aass, et al.^16^ reported a reduction of 10% in the variance of the predicted IMF compared to the observed IMF using ultrasound measurements in lean pure and crossbred beef and dual-purpose bulls and steers (2.01 ± 1.52%; means ± SD). Further, the predicted IMF was only 0.2% below and above the selected range. Therefore, our study concludes that the prediction of IMF using generalized additive model and lauric acid in hair as predictor shows good performance, especially in an IMF range that is important to reach acceptable palatability of the beef.

Additionally, the reason for the moderate correlations achieved could lie in the origin of fatty acids in hair. In humans, sebaceous glands, blood circulation, or the follicle itself are reported as sites of synthesis^17^.The predominant fatty acids in the LT muscle were also found in the hair of crossbred bulls. While the IMF content in LT consists of equal proportions of SFA and MUFA, hair fat mainly composes of SFAs. The high proportion of SFA in the hair is mainly driven by the *de novo* synthesized fatty acids capric, lauric and myristic acid. Hair from crossbred bulls taken one to four days before slaughter have 1.5-times higher myristic acid (%) and 0.7-times lower palmitic and stearic acid (%) compared to hair from cows of the dairy cattle breed Holstein or dual-purpose breed Simmental taken 8 weeks after calving^12^.

Our study found that under the same feeding and husbandry conditions on farm 2, the oleic acid percentages in LT compared to hair were 1.1- and 2.5-times higher in UCKxBEEF than WBBxSBT bulls, respectively. As UCKxBEEF and WBBxSBT bulls are genetically very different crossbreeds, the effect of breed on fatty acid composition was expected and had been reported before^18,19^. In our study we show for the first time that a high availability of oleic acid in the metabolism of bulls is not only mirrored by a high IMF content in LT, but also by a high oleic acid percentage in the hair. Different to oleic acid, the percentages of palmitic and stearic acid in LT and hair were at the same level. While the group sizes on farm 2 were unbalanced, we propose to investigate the potential of hair fatty acid profiles to identify the genetic predisposition to high and low IMF in finishing bulls under the same feeding and husbandry conditions in further studies.

Besides the positive correlation between IMF content in LT and MUFA (oleic acid) in the hair, such a positive correlation was also found between the IMF content in LT and percentages of odd and branched chain fatty acids and PUFAs in hair. As an essential fatty acid, linoleic acid is the major PUFA in ruminant diets. Under equal feeding and husbandry conditions on farm 2, linoleic acid (%, mg/ 100 g) was 2.2- to 2.9-times higher in hair from UCKxBEEF compared to WBBxSBT. Recently, a study in early lactating cows has shown that a higher linoleic acid percentage in hair mirrors higher energy intake^12^. Since higher energy intake leads to higher IMF storage^20^, we suggest that bulls with a higher IMF content in this study had also a higher energy intake, even if no individual measurement of energy intake was performed.

## Conclusion

To our knowledge, the present study was the first approach to investigate the relationship between IMF content of muscle after slaughter and the fatty acid composition in hair taken before slaughter, with the aim of selecting potential markers for decisions on finishing fattening of beef bulls to improve meat quality. The advantage to use the hair as a tissue is that it can be taken non-invasively and easy from live animals which makes an application on production farms feasible. The study provides evidence that lauric and oleic acid in hair taken shortly before slaughter provide significant information about the IMF content. Additionally, our results suggest that under same feeding and housing conditions crossbreed-specific differences occur for capric, lauric, myristic and also oleic acid in the LT and in the hair. The analysis of the fatty acid composition of hair extends the non-invasive methods to assess the IMF content in vivo. For influencing the IMF content e.g., by prolonged feeding or changing the fattening strategy, it would be necessary to obtain the information of fatty acids in the hair early enough before the decision of slaughtering. Therefore, it would be necessary to extend the current study by testing earlier time points of hair sampling before slaughtering.

## Materials and methods

### Animals

The study was carried out on two typical farms for the finishing of bulls in the north-east of Germany. On each farm, a group of bulls that had completed the finishing period at the same time was randomly selected. The bulls were slaughtered at the same commercial slaughterhouse in Teterow, Germany, which is managed by Danish Crown (Randers, Denmark). The distance to the slaughterhouse was 116 km and 30 km from farm 1 and 2, respectively. All animals were slaughtered within 1 h of their arrival. Farm 1 had 21 UCK x BEEF bulls (from 10 purebred sires of Uckermärker (UCK) and 21 dams which are crossbreeds between UCK and Simmental, Charolais, or Limousin (BEEF)). Farm 2 had 15 UCKxBEEF bulls (from 7 UCK sires and 15 BEEF dams) and 8 WBBxSBT bulls (from 3 Belgian Blue (WBB) sires and 8 German Schwarzbunte Holstein (SBT) dams). All WBBxSBT bulls were heterozygous for myostatin because all WBB sires were homozygous myostatin mutation carriers. The exact pedigree was unknown in both farms. All bulls were housed and cared for according to the German animal welfare act^19^. As the hair samples were taken non-invasively, the study was not categorized as an animal experiment.

The bulls were purchased at six months of age on both farms and were kept in groups of 7 to 8 animals (farm 1) and 5 to 6 animals (farm 2) until slaughter. The bulls were fed a farm-specific diet (see Supplemental material Table 7). Bulls had *ad libitum* access to feed and water. Bulls were selected for slaughter by the farm manager when they were between 17 and 24 months old.

### Hair sampling and analysis of fatty acid composition of the hair

A hair sample per bull was taken from a dorsal region of the back between the 5th and 6th rib, 4 days *ante mortem* on farm 1, and one day *ante mortem* on farm 2. The shaved area was 80 cm² big and the hair cut was performed with a clipper (Adelar Pro, WAHL, Georgen, Germany) and a blade (Moser Magic Blade Super Fine, WAHL, Georgen, Germany) with a cutting height of 0,5 mm (without hair follicle). The hair sample was taken directly above the locus where the LT sample was taken for IMF and fatty acid analysis. Sampling was conducted in November 2021 and April 2021 on farms 1 and 2, respectively. The hair samples were stored at -20 °C until the analysis of fatty acids.

### Determination of intramuscular fat content

To access the LT, the 6th rib (cranial) was removed from the left side of the carcass 18 h after slaughter at the industrial abattoir and transported 40 km to the meat quality laboratory at the Research Institute for Farm Animal Biology in Dummerstorf, Germany. Afterwards, the LT was removed and cut into a 3 cm thick steak. This steak was homogenized to determine the total IMF content with near-infrared spectrometry using the FoodScanTM Lab (Foss, Hillerød, DK). One sub-sample of the homogenized raw meat was used for analyzing the fatty acids in LT.

### Fatty acid analysis of hair and muscle

The fatty acid composition of the LT and hair samples were determined using the established and published protocols by^22,23^. Hair lipids were extracted from 200 mg of cleaned, homogenized and complete mill-ground hair, muscle lipids from 1 g homogenized raw meat. For the quantification of fatty acids, C19:0 was used as an internal standard. Fatty acid transmethylation was performed using sodium methoxide in methanol and 14% boron trifluoride in methanol. Fatty acid methyl esters were extracted twice with 2 mL *n*-hexane. Final extracts were not evaporated in order to retain the slightly more volatile fatty acids, such as lauric acid, and stored at -18 °C until high-resolution gas chromatography analysis. The determination of fatty acid methyl esters of LT and hair was carried out using the same PerkinElmer gas chromatograph CLARUS 680 with a flame ionization detector and split injection (PerkinElmer Instruments, Waltham, Massachusetts, USA) equipped with a CP-Sil 88 CB column (100 m × 0.25 mm, Agilent, Santa Clara, CA, USA). Although the capillary high-resolution gas chromatography was the same for the analysis of hair and LT sample extracts, the temperature program was tissue specific as reported previously^22,23^. All samples were analyzed twice. Across both farms, 22 and 39 fatty acids were reliably detected in hair and LT, respectively.

### Statistical analysis

Statistical analyses were performed using R 4.0.2^24^. The Wilcoxon test was used (1) to test the farm-specific differences of performance data, IMF content and fatty acid profiles of LT and hair between UCKxBEEF from farm 1 and from farm 2 and (2) to evaluate crossbreed-specific differences within farm 2 between UCKxBEEF and WBBxSBT. Differences of fatty acid composition between LT and hair were analyzed within each farm using an analysis of variance for repeated measurements including tissue (LT and hair) as fixed effect. Since multiple measurements were available of each bull, the bull was included as repeated measurement. The results were expressed as estimated marginal means (EMM) and standard error of the mean (SEM). Additionally, Spearman’s correlation analyses were performed to assess whether fatty acids in hair shortly before slaughter show a correlation to individual fatty acids and the sum of fatty acids in LT as well as to IMF content of the muscle. The Spearman correlation was chosen because the hair fatty acids were not normally distributed. The bulls in farms 1 and 2 had different genetic backgrounds. In farm 1, only UCKxBEEF bulls were housed, in farm 2 bulls from the two crossbreeds UCKxBEEF and WBBxSBT. Consequently, the analyses were carried out for 4 groups: across all animals (2 farms, 2 crossbreeds), across the UCK x BEEF bulls from farm 1 and 2 and across WBBxSBT from farm 2. Only significant correlations are presented. The significance threshold was set at *P* ≤ 0.05.

Individual and sums of fatty acids in hair (% of total fatty acids; mg/ 100 g tissue) that shown significant correlation to IMF (%; Supplemental material Tables 5 and 6) were used as predictors to develop IMF prediction models. Since it is not yet known which model is best suited for predicting IMF (%) based on hair fatty acids, we tested linear regression as linear approach (base R package; function: lm), quantile regression as non-parametric approach (R package: quantreg; function: rq), as well as generalized additive model (R package: gam; function: GAM) and randomForest (R package: randomForest; function: randomForest) as non-linear approaches. Each model was built with farm, crossbreed and one predictor fatty acid. To verify prediction performance, the mean square error (MSE; %) was calculated to assess the prediction error of the model, and the Spearman correlation coefficient (r_S_) to evaluate the rank-based association between predicted and observed values while applying the leave-one-out method. In this method, each bull was used as a test set, while the remaining bulls were used as trainings set (n-1). Only fatty acids in hair that showed a consistent ability to predict IMF in live bulls in the tested models are reported. In this study, consistency was defined as the highest correlation coefficients between observed and predicted IMF and lowest MSE across all four modeling approaches tested. Due to the low sample size (44 bulls), we additionally check the best model for agreement between the observed and predicted IMF as well as the confidence interval in an interesting range of IMF between 3.5 and 4.5% to reach acceptable palatability.

## Supplementary Information

Below is the link to the electronic supplementary material.

Supplementary Material 1

## Data Availability

The data presented in this study are available on reasonable request from the corresponding author.
